# Floral Development at Multiple Spatial Scales in *Polygonum jucundum* (Polygonaceae), a Distylous Species with Broadly Open Flowers

**DOI:** 10.1371/journal.pone.0102802

**Published:** 2014-07-24

**Authors:** Lan-Jie Huang, Wen-Long Fu, Xiao-Fan Wang

**Affiliations:** College of Life Sciences, Wuhan University, Wuhan, China; Central China Normal University, China

## Abstract

Distyly, a special polymorph, has evolved in many groups of angiosperms and has attracted attention since Darwin’s time. Development studies on distylous taxa have helped us to understand the evolutionary process of this polymorph, but most of these studies focus on species with narrowly tubular corolla. Here, we studied the floral development of *Polygonum jucundum*, a distylous species with broadly open flowers, at multiple spatial scales. Results showed that the difference in stigma height between flowers of the two morphs was caused by differences in style growth throughout the entire floral development process. The observed difference in anther heights between the two morphs was because the filaments grew faster in short-styled (SS) than in long-styled (LS) flowers in the later stages of floral development. In addition, the longer styles in LS flowers than in SS flowers was because of faster cell division in the early stages of floral development. However, SS flowers had longer filaments than LS flowers primarily because of greater cell elongation. These results indicate that floral development in *P. jucundum* differs from that of distylous taxa with floral tubes shown in previous studies. Further, we conclude that the presence of distyly in species with open flowers is a result of convergent evolution.

## Introduction

Heterostyly is a unique polymorph of the reproductive structure in plants, a phenomenon that has no parallel in the animal kingdom [1], [2]. Heterostylous species could be divided into distylous and tristylous taxa. The distylous taxa consist of long-styled (LS) flowers with stigmas above the anthers and short-styled (SS) flowers whose stigmas are below the anthers. The tristylous taxa consist of three morphs, in which the stigma-height of each morph differs from the other two [1], [3]–[5]. The polymorphic structure in heterostylous species is designed to improve the accuracy of pollen transfer between different morphs, thus, ensuring the outbreeding of these species [5], [6]. In 28 families with heterostyly, the distylous morph is more prevalent and has recently attracted great attention from botanists [2], [4], [7]–[12].

The evolution of distyly could progress through two different patterns. It is thought that either the dimorphic height of floral organs evolves after the appearance of self-incompatibility in homostylous ancestor [13] or distylous taxa evolve from a self-compatible ancestor with a herkogamous floral morphology [6]. The evolutionary process of some distylous taxa has been revealed by investigations into the development of floral organs [10], [14]–[16]. In the floral development of these distylous species, four different development pathways are known to cause differences in stigma heights between LS and SS flowers. The difference in style heights is because of divergence in style growth during early or late stages of bud elongation. Only two different development pathways are observed in the divergency of anther heights between two types of flowers. The relative growth rate of buds is very important in these two pathways [10], [16]–[19]. These developmental differences suggest that a stigma-height mutation is the probable cause of distyly and that the ancestors of these distylous taxa have “approach herkogamy” or “reverse herkogamy” with stigmas borne at a different level to the anthers [10], [16].

Although these floral development studies have helped us to understand the evolution of floral dimorph, they are limited to families that contain a corolla tube to which anthers adhere through a short filament [10], [14], [20]–[25]. However, some distylous flowers in other families are broadly open rather than narrowly tubular [26]. The anther heights in these flowers have no direct relationship with the bud length. Thus, the floral development processes and the dimorph evolution in these taxa may differ from the taxa with floral tubes investigated in previous studies.

*Polygonum* is a widely distributed genus belonging to the Polygonaceae family [27], [28]. Heterostyly was recorded in this genus over 100 years ago [1]. There are over 300 species within the *Polygonum* genus, and scholars have studied its floral dimorph since 1977 [27], [29], [30]. However, no information on the floral development and evolution of these distylous species has been reported. The tepals in the flower of *Polygonum* are separate while two whorls of stamens are borne at the floral base near the ovary. In this study, we investigate the floral development of *Polygonum jucundum* at multiple spatial scales in order to answer the following questions: (1) how does floral-organ development differ between LS and SS flowers in *P. jucundum*; (2) how do histological processes influence this macroscopic difference; and (3) what is the probable evolution pattern of the dimorph in this distylous species.

## Materials and Methods

### Ethics Statement

i. Specific permission was not required for study at our site;

ii. Endangered or protected species do not grow in the study site.

### Study species

**Figure 1:**
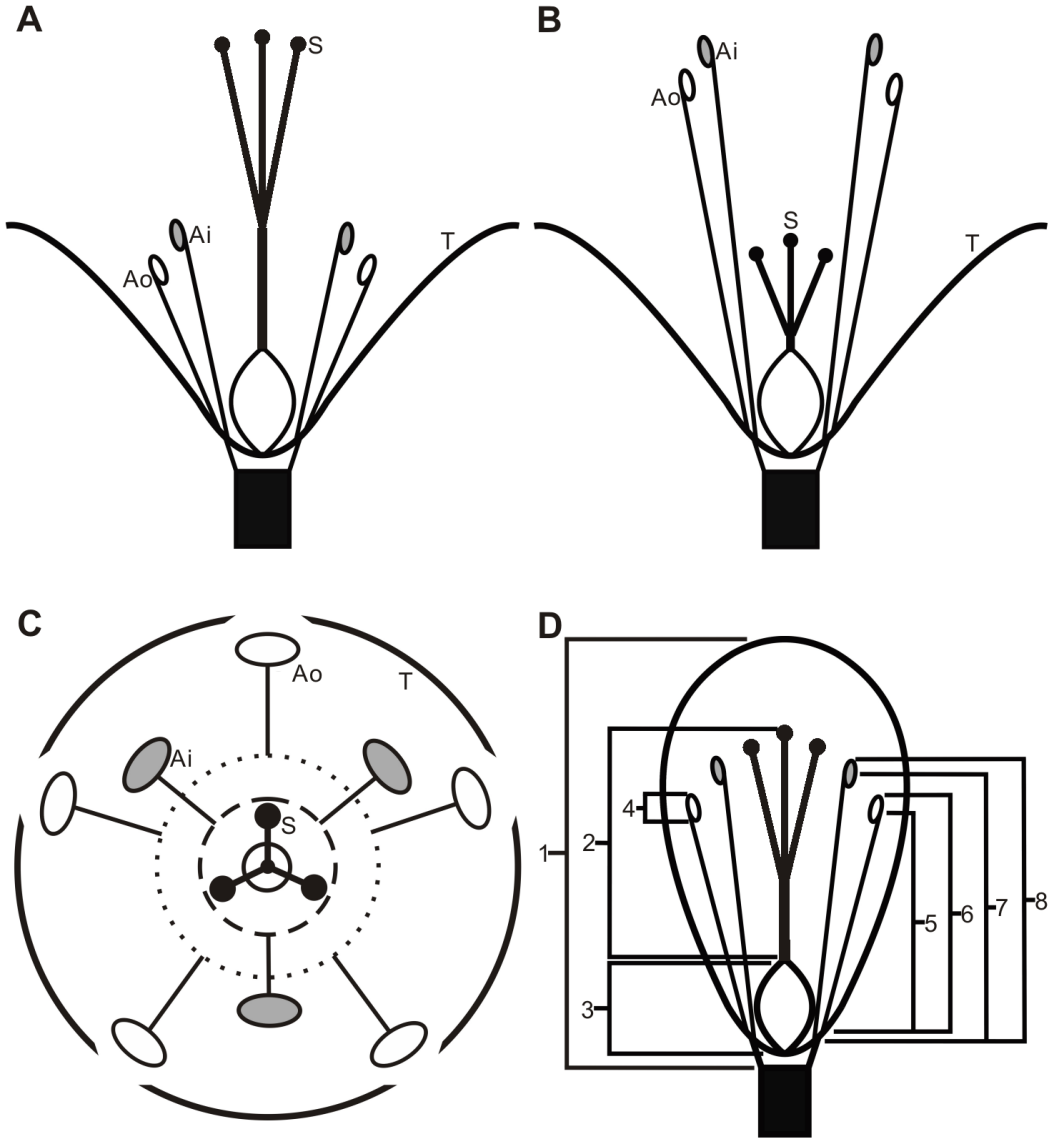
The structure of flower and floral organ measurements in *Polygonum jucundum*. A, the longitudinal section of the long-styled flower; B, the longitudinal section of the short-styled flower; C. the cross section of the flower; D, floral organ measurements: (1) bud length, (2) style length, (3) ovary length, (4) anther length, (5) outer filament length, (6) outer anther height, (7) inner filament length, (8) inner anther height. Ai, inner anther; Ao, outer anther; S, stigma; T, tepal.

*P. jucundum* is an annual, diffuse herb, which is widely distributed in southern China [27]. It is morphologically distylous and possess broadly open flowers (Fig. 1A, B), with some intra-morph compatibility. In these flowers, anthers and stigmas are reciprocally positioned in three dimensions. In addition, there are eight stamens at the base of separate tepals in two whorls; a pistil with one ovary and three stigmas borne in the floral center (Fig. 1C). Unlike in tubular distylous flowers, the anther height is determined by filament length and anther length while the stigma height is constituted by style length and ovary length. A previous study revealed that flowers of SS individuals have slightly shorter tepals, fewer pollen grains, and larger stigmatic surfaces. By contrast, LS flowers produce larger quantities of pollen grains that are smaller in size [27].

Plant material of *P. jucundum* was collected from a wild population in the suburb of Wuhan, Hubei Province, China (30°31′N, 114°29′E). The sampling strategy was designed to ensure adequate representation of the entire morphological range for this species. All experiments were carried out during November and December 2012 and 2013.

### Microscopy and Measurements

For observations and measurements on floral organs in *P. jucundum*, two inflorescences with buds at similar stages of development were selected from each of 10 LS and 10 SS plants. The size of buds in these inflorescences was measured from early developmental stages until anthesis, and these data were used as a relative estimate of developmental time according to Richards and Barrett [32]. The beginning of floral development (metrical origin) was defined as that stage of development when buds were about 0.5 mm in size.

A hundred and forty-four buds from each morph range from metrical origin to blooming were dissected and observed using an Olympus SZX2-ILLT stereomicroscope. Tepals were carefully removed from each bud by using a dissecting needle. Stamens and pistil on the receptacle were flattened on a glass slide. Images of floral organs were captured using an Olympus digital camera. The ovary length, style length, anther length, outer anther height, inner anther height, outer filament length, and inner filament length were measured from these images by using the metrical software ImageJ (Fig. 1D).

To study the cellular variation during floral development, 13 buds from each morph, representing the various stages of development, were dissected. Whole filaments and styles in each bud were picked and laid on a glass slice with fast green dye for about 1 min. The dyed material was then covered using a coverslip and observed under an Olympus BX 43 light microscope. Whole filaments and styles were photographed using an Olympus digital camera while focusing on the epidermal cells. Along a whole filament or style, the cell numbers in a single cell column were recorded. These measurements were taken 10 times on each of the inner filament, outer filament, and style in a flower. In addition, 100 epidermal cells were chosen randomly in each type of floral organ per flower and their lengths were measured using the metrical software ImageJ.

### Data Analysis

In order to understand the developmental process of floral organs within a bud, outer anther height, inner anther height, and stigma height were compared across different bud lengths in which stigma height equaled style plus ovary lengths. These comparisons were made in each morph, and the results were fitted to regression equations. In order to identify the major factor causing differences in floral organ heights between flowers of the two morphs, LS and SS floral buds were compared by plotting the bud length against ovary, anther, inner filament, outer filament, or style lengths. Plotted data were fitted to linear or quadratic regression equations depending on scatter patterns. The slopes of the lines generated for LS and SS flower buds were compared using analysis of covariance (ANCOVA) homogeneity of slopes model. The mean cell number and cell length of inner filament, outer filament, or style in each flower were calculated. The data for LS and SS flowers were reported by bud length. Trend lines were plotted for each group of data. All plots and statistics were obtained using SPSS 17.0 and SigmaPlot 12.0.

## Results

### Comparison of reproductive organ heights within a flower during development

**Figure 2:**
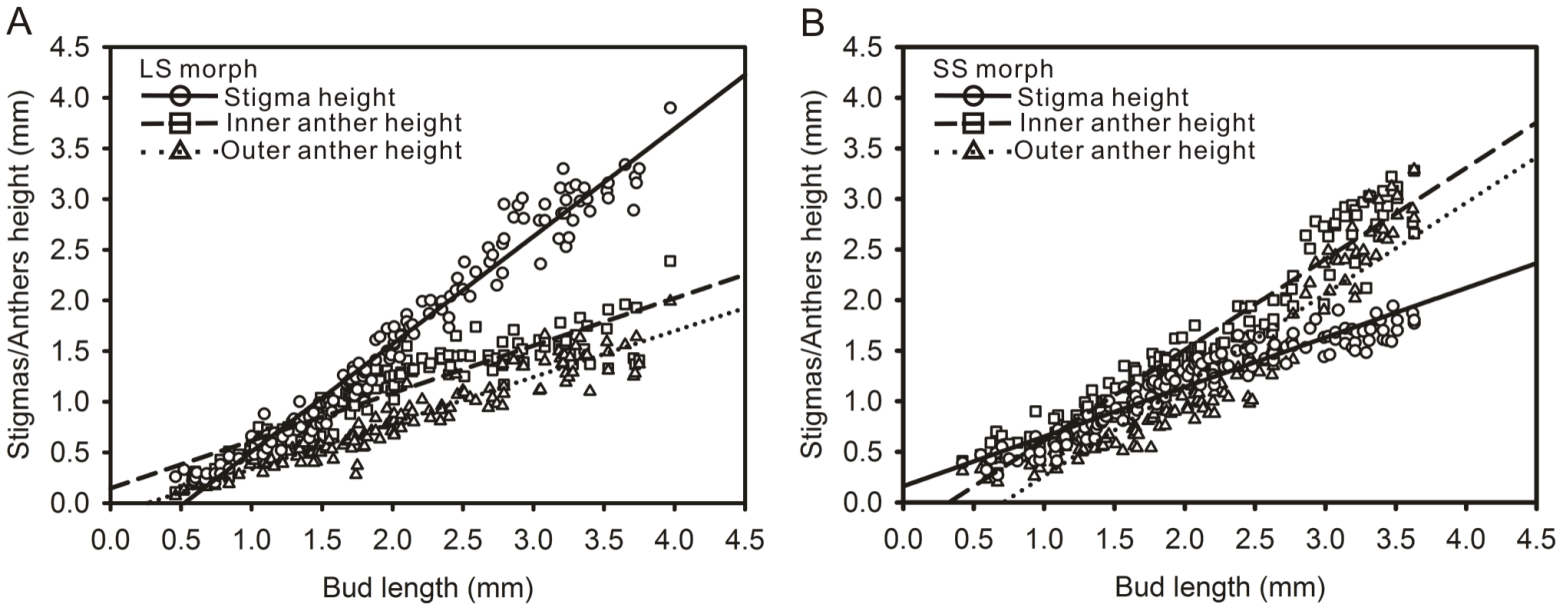
Floral organ heights in flowers of two morphs during floral development of *Polygonum jucundum*. A, the long-styled flower; B, the short-styled flower. Regression lines were fitted separately to data for each floral organ.

Stigma and anther heights were assessed to determine the difference in the developmental process among the reproductive organs in a flower. In the earliest stages of bud elongation, the heights of all reproductive organs in a flower were nearly identical (Fig. 2). The difference between anther height and stigma height became obvious when the bud length approached 2.0 mm. After blooming, the stigma was about twice the height of the anther in LS flowers; however, in SS flowers the anther was about 1.5 times taller than the stigma (Fig. 2). Although the inner stamens were taller than the outer stamens throughout the whole development process (Table 1),the difference of the growth rate between two whorls of the stamens was not significant (P_LS_ = 0.480, P_SS_ = 0.968).

**Table 1:** Relationship between floral organ height and bud length in long-styled (LS) and short-styled (SS) flowers of *Polygonum jucundum.*

Floral morph	Floral organ	Sample size (plants, flowers)	Regression		
			R^2^	Slope	Intercept
LS (P^1^<0.0001, P^2^ = 0.480)	Stigma height	(10, 144)	0.96	1.063***	−0.556
	Inner anther height	(10, 143)	0.85	0.469***	0.147
	Outer anther height	(10, 143)	0.91	0.454***	−0.120
SS (P^1^<0.0001, P^2^ = 0.968)	Stigma height	(10, 144)	0.90	0.490***	0.161
	Inner anther height	(10, 143)	0.93	0.899***	−0.291
	Outer anther height	(10, 142)	0.88	0.900***	−0.640

Differences between regression slopes for each floral organ are indicated in parentheses after the morph illustration.

1 Stigma vs Anther;

2 Inner anther vs Outer anther;

***P<0.0001.

### Length of floral organs in LS and SS flowers

Ovary length plus style length constituted the stigma height while anther length plus filament length determined the anther height. All these measurements were used to compare LS and SS flowers in order to determine the factors responsible for the difference in height of reproductive organs.

**Figure 3:**
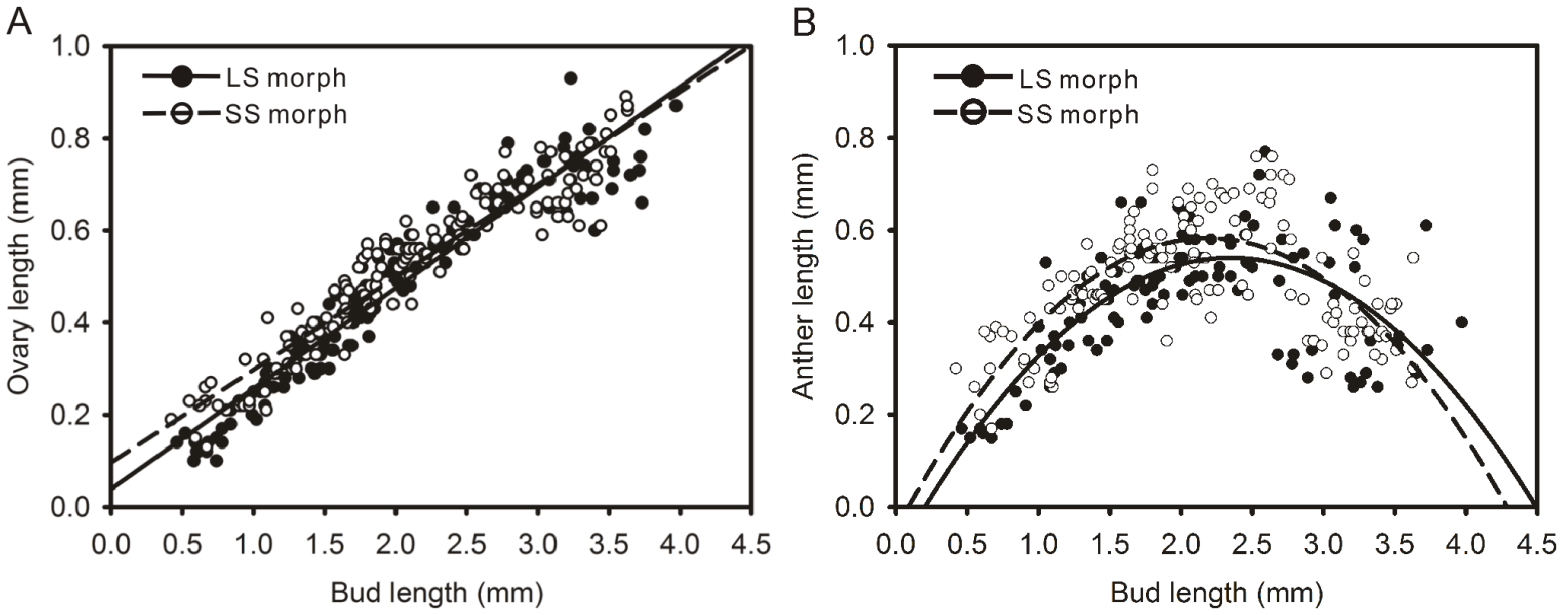
The development of ovaries and anthers in two morphs of *Polygonum jucundum*. A, Ovary; B, Anther. Regression lines were fitted separately to the data for each floral morph.

The growths of the ovary and anther during the entire floral development in the two morphs were very similar (Fig. 3). The linear term explained most of the variation in the relationship between bud length and anther length in both LS and SS flowers (Table 2). In both morphs, the slope based on linear regression for bud length on anther length explained less than 10% of the variation (Table 2). However, a significant second-order term improved the predictive fit of growth patterns to more than 50% of the variance (Table 3). Thus, the relationship between growth of anther and bud elongation was curvilinear. From the quadratic fitting curves in Figure 3, we found that the maximum length of anthers during stamen growth differed slightly between the two morphs. The maximum anther lengths in SS and LS flowers were closer to 2.0 mm.

**Table 2:** Linear regression of relative growth rates of floral organs against bud length for long-styled (LS) and short-styled (SS) flowers of *Polygonum jucundum.*

Floral organ	Floral morph	Sample size (plants, flowers)	Linear Regression		
			R^2^	Slope	Intercept
Ovary (P = 0.029)	LS	(10, 144)	0.91	0.218***	0.039
	SS	(10, 144)	0.91	0.201***	0.097
Anther (P<0.0001)	LS	(10, 110)	0.08	0.046**	0.354
	SS	(10, 144)	0.01	0.016	0.453
Style (P<0.0001)	LS	(10, 144)	0.95	0.845***	−0.595
	SS	(10, 144)	0.83	0.289***	0.064
Inner filament (P<0.0001)	LS	(10, 142)	0.85	0.434***	0.022
	SS	(10, 143)	0.92	0.892***	−0.488
Outer filament (P<0.0001)	LS	(10, 142)	0.83	0.878***	−0.837
	SS	(10, 142)	0.89	0.436***	−0.273

Differences between regression slopes for each floral morph are indicated in parentheses after the organ name.

***P<0.0001;

**P<0.01.

**Table 3:** Quadratic regression of relative growth rates of floral organs against bud length for long-styled (LS) and short-styled (SS) flowers of *Polygonum jucundum.*

Floral organ	Floral morph	Polynomial Regression (f = y0+a*x+b*x∧2)			
		R^2^	y0	a	b
Ovary	LS	0.94	−0.120	0.397	−0.042***
	SS	0.92	−0.003	0.312	−0.026***
Anther	LS	0.51	−0.107	0.551	−0.117***
	SS	0.55	−0.049	0.578	−0.132***
Style	LS	0.96	−0.353	0.573	0.063**
	SS	0.87	−0.244	0.634	−0.081***
Inner filament	LS	0.88	−0.360	0.858	−0.098***
	SS	0.95	0.238	0.080	0.191***
Outer filament	LS	0.91	−0.017	0.152	0.065***
	SS	0.94	0.654	−0.779	0.389***

Differences between regression coefficients for each floral morph are indicated in parentheses after the organ name.

***P<0.0001;

**P<0.01.

**Figure 4:**
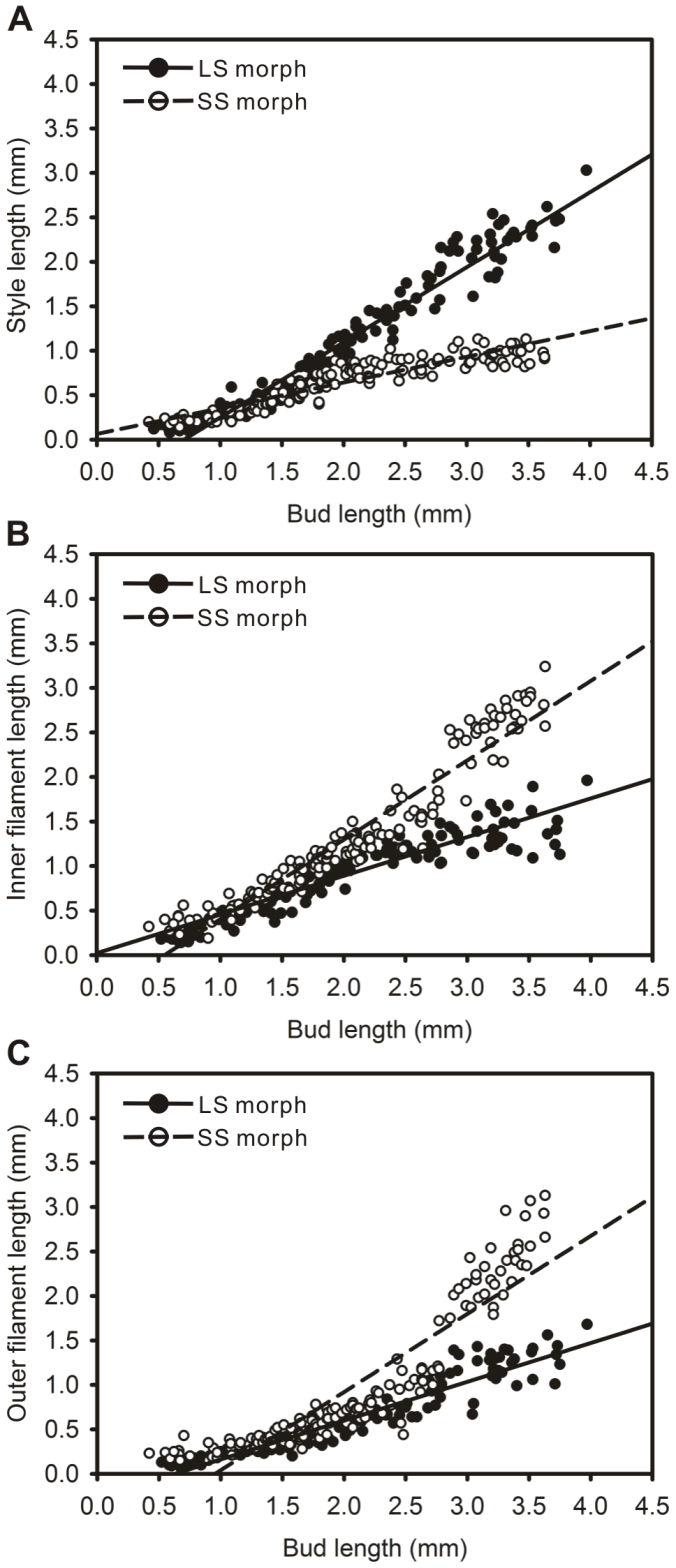
The development of styles and filaments in two morphs of *Polygonum jucundum*. A, Style; B, Inner filament; C, Outer filament. Regression lines were fitted separately to the data for each floral morph.

The difference in style or filament growth between LS and SS flowers in the development process was very significant (P<0.0001). The linear term explained most of the variation in the relationship between bud length and style length or filament length in both morphs (Table 2). However, some small improvement occurred when a second-order term was added (Table 3). This indicates that the relationship between bud elongation and growth of style or filament length has some curvilinear characteristics. Starting from the early stage of development, the style in LS flowers grew faster than in SS flowers (Fig. 4A). Filaments in the flowers of the two morphs elongated at similar speed when bud length was less than 2.5 mm. When bud length was greater than 2.5 mm, filaments in SS flowers grew faster than in LS flowers (Fig. 4B, C). Both whorls of filaments displayed a fluctuating growth rate (Fig. 4B, C).

### Variation of epidermal cells in the developmental process of floral organs

**Figure 5:**
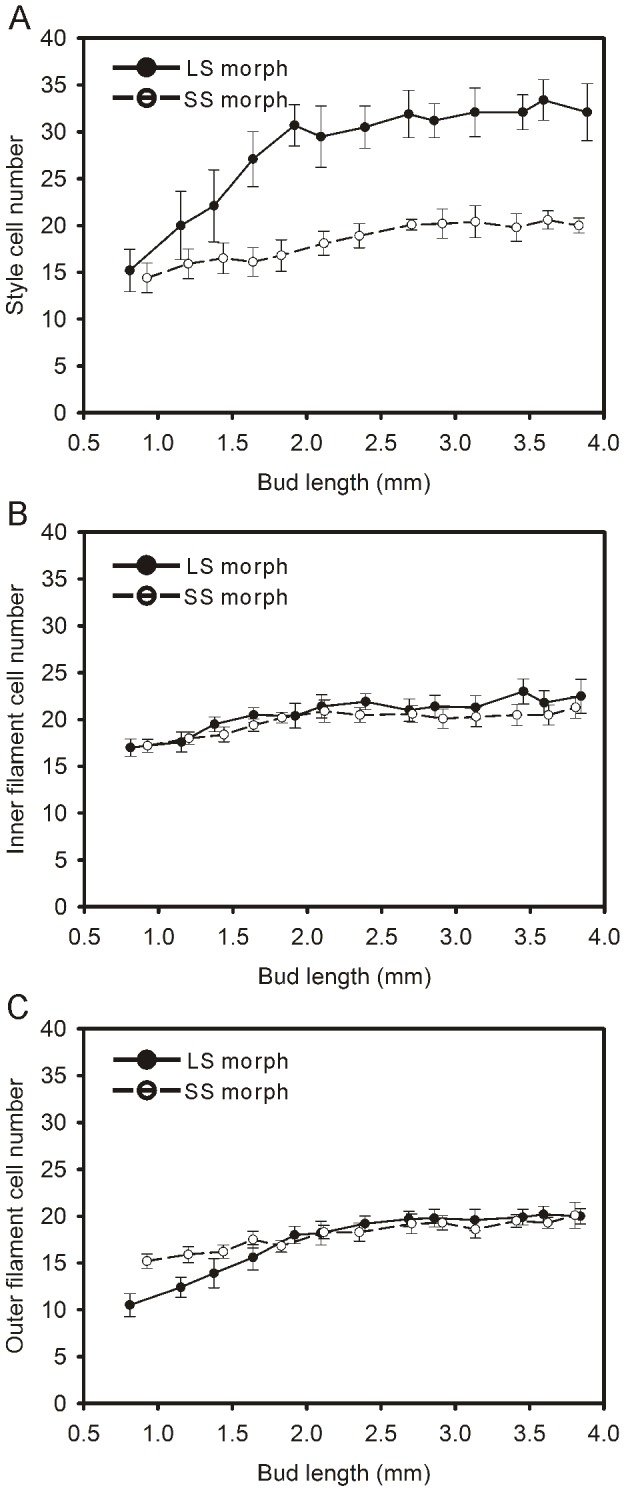
The number of epidermal cells in a single cell column along styles or filaments during the floral development of *Polygonum jucundum*. A, Style; B, Inner filament; C, Outer filament. Trend lines were plotted separately for the data for each floral morph. Error bars indicate counting errors.

The growth of the filament or style is influenced by cell division and elongation. The variation in the number and length of epidermal cells in these organs during bud growth could explain the developmental patterns at the microscopic scale. There was a small increase in the number of epidermal cells in the style of SS flowers when bud sizes changed from 0.5 mm to 4.0 mm. The number of epidermal cells in the style of LS flower increased sharply from the early stage of floral development, reaching a maximum when bud size was at 2.0 mm. At bud sizes greater than 2.0 mm, no further increase in cell numbers was observed up to anthesis (Fig. 5A). The maximum number of epidermal cells in LS flowers was more than 1.5 times greater than in SS flowers (Fig. 5A). There was little change in cell numbers in the inner filament and outer filament as bud length increased (Fig. 5B, C). The cell numbers did not differ between the two morphs.

**Figure 6:**
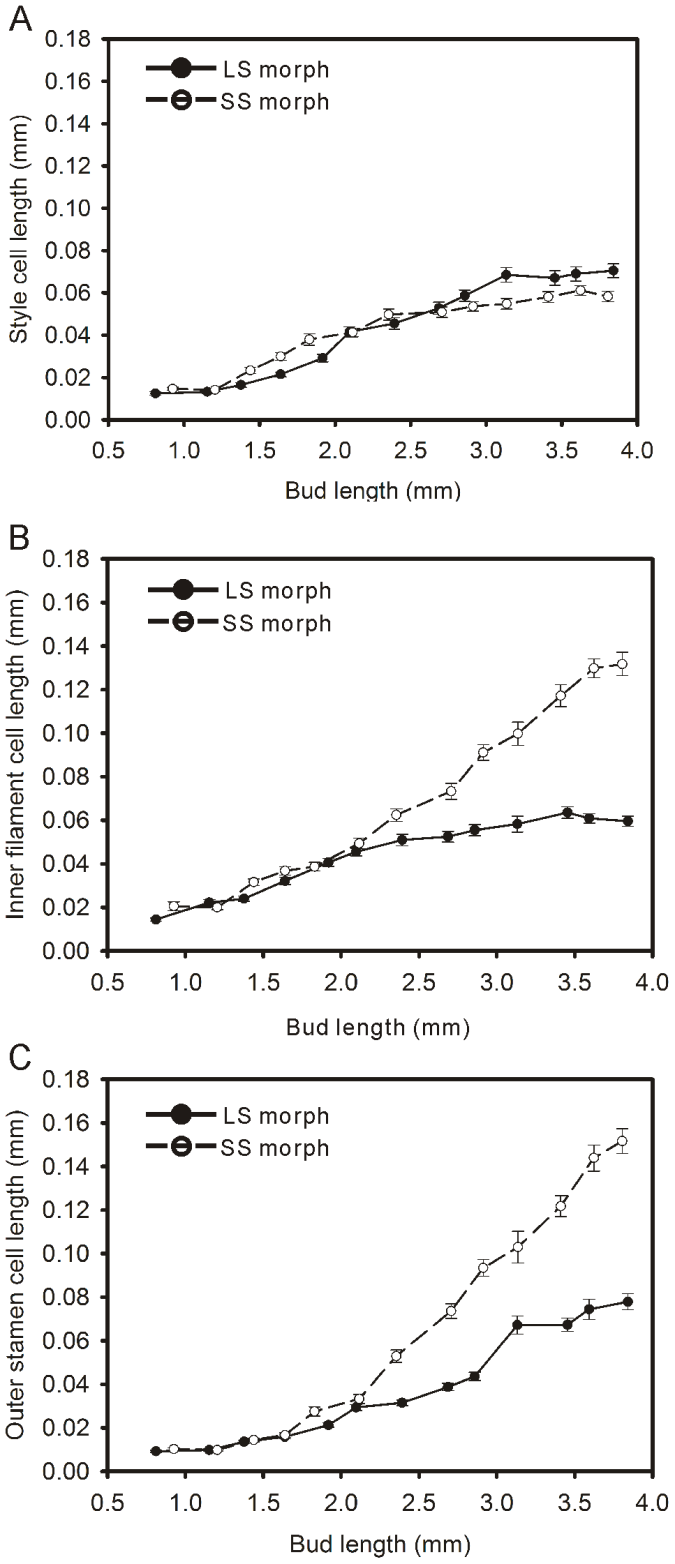
The lengths of epidermal cells in styles or filaments during floral development of *Polygonum jucundum*. A, Style; B, Inner filament; C, Outer filament. Trend lines were plotted separately for the data for each floral morph. Error bars indicate the range in epidermal cell lengths.

The growth rate of epidermal cells in the style was constant throughout the floral development process. This growth rate did not differ between LS and SS flowers (Fig. 6A). In the inner filament, cell elongation followed the same pattern in the two morphs until the bud size reached 2.5 mm long. At this point, cells in SS flowers elongated faster than in LS flowers. When flowers had completely opened, the length of cells in SS flowers was twice that of cells in LS flowers (Fig. 6B). Cell growth in the outer filament resembled that of the inner filament, but from the photographs, we found that cells of outer filament grew faster than did those in the inner filament for both LS and SS flowers (Fig. 6C).

## Discussion

### Floral developmental patterns in *P. jucundum*

Previously studied distylous taxa had narrow tubular corolla and the filament served only to attach the anther to the inner surface of the corolla tube. Thus, the filament made little contribution to the anther height in these species [10], [16], [31]. However, in *P. jucundum*, the stamens are independent from the separate tepals and they possess long filaments. There are obvious differences between the anther growth patterns in the flowers of the two morphs. However, the anther length is much shorter than the filament length. Anther height is mainly determined by the filament length. The divergence of anther height between LS and SS flowers occurs in the later stages of bud elongation. This finding is in contrast with observations in species of Rubiaceae and Boraginaceae where divergence was found to occur in the early stages [10], [18]. In addition, the anther heights in two whorls of stamens are not isometric at all times, which is probably a result of inner stamens being initiated earlier than the outer stamens.

Although the pistil of *P. jucundum* possesses three somewhat separate stigmas, the stigma heights vary consistently with the bud growth. During floral development, the length of the ovary in the pistil increases at a constant speed related to the bud length. The difference in ovary elongation between LS and SS flowers was very small. Considering that stigma length is negligible, the difference in stigma height between flowers of two morphs is primarily determined by differences in style length. LS flowers had higher stigmas than those in SS flowers because of the faster growth rate of the style in LS flower, which occurred in the early stages and was maintained throughout flower development. This has also been observed in some species of Rubiaceae [10], [19].

Cell division and cell elongation could contribute to the growth of floral organs [11], [16], [20]. Different microscopic patterns of floral development were found in some distylous species with tubular corolla, which showed cell division mainly served to influence anther height in two morphs. The different gynoecium growth in both morphs was affected by both cellular fission and extension [16]. However, in *P. jucundum*, during style growth, cell elongation in LS morph was similar to that in SS morph, but cell division in LS morph was greater than in SS morph. This difference in cell division was observed in the early stages of floral development. The difference in cell numbers and the sustained elongation of the cells resulted in styles of different lengths between the two morphs, which continued to increase until blooming. However, cell numbers in the filaments of mature flowers of the two morphs are similar. This suggests that the difference in filament length is entirely due to differences in the process of cell elongation.

Contrary to observations in *Lithospermum* [16], differences in stigma height between the two morphs of *P. jucundum* were recorded in the early flower development stages while the difference in anther height is evident in the later stages. This could also be explained by histological processes in floral development. In both the style and filament, the process of cell division ends in the early phase. As a result, LS flowers, which possessed stronger cell division in the style, have higher stigmas than SS flowers in the early stages of development despite the shorter cell lengths. However, because the processes of cell division in the filament between the two morphs are similar, and there is little cell growth in filament in the early phases, anther heights do not differ much in the early development stages. However, when the bud length approached 2.5 mm, SS and LS filament cells grew at different rates resulting in differences in anther height between the two morphs.

The bud length of 2.5 mm was achieved shortly after the anther length had reached its maximum, which is probably not coincidental. Scholars have previously indicated that cell elongation in filaments could be affected by auxins produced by the anthers [33],[34]. At different developmental stages, the anther produces pollen of different sizes and numbers and could also produce different levels of auxins [34]. The anther length reaches the maximum shortly after meiosis of sporogenous cells, while auxins accumulated in the anther also approach the maximum level [35]. This maximal level is probably different in LS and SS flowers because of different number or size of sporogenous cells and affects the cell elongation of two morphs differently [36].

### The evolution of distyly of *P. jucundum*

Few studies have documented floral development and evolution in distylous taxa with broadly open flowers [2]. Our study in *P. jucundum* reveals a detailed development process in floral organs of distylous taxa at multiple spatial scales, which is different from the processes reported in distylous taxa with narrowly tubular corolla. Although we are not able to declare that all distylous taxa with open flowers have a floral development process similar to that of *P. jucundum*, we are convinced that distyly in some species with open flowers could be a result of convergent evolution, as seen in *P. jucundum.*

*Polygonum* consists of many homostylous species and some distylous species [16], [37]. These distylous species probably possess a homostylous ancestor and seem to evolve according to the model presented by Charlesworth and Charlesworth, in which self-incompatibility arose prior to the morphological features of distyly [13]. However, some studies indicate that it is self-compatibility in both homostylous and distylous species of *Polygonum* [37]. In another model proposed by Lloyd and Webb, the morphological features preceded the evolution of an incompatible system [6]. In addition, the approach that distylous taxa have a herkogamous ancestor is needed in this model [6]. A first mutation that reverses stigma height and a second mutation that adjusts anther height are considered to occur in the population of these ancestors, producing a reverse-herkogamous morph, which can become established in the population because of a frequency-dependent advantage in pollen transfer [38], [39]. The difference in stigma height between the two morphs, which preceded differences in anther height during floral development of *P. jucundum*, probably suggests that distyly in *P. jucundum* evolved according to the pattern proposed by Lloyd and Webb [6]. To prove this, further research on development and phylogenetic of distyly in *Polygonum* and other species of Polygonaceae family is required.
